# Sox2 and Pax6 Play Counteracting Roles in Regulating Neurogenesis within the Murine Olfactory Epithelium

**DOI:** 10.1371/journal.pone.0155167

**Published:** 2016-05-12

**Authors:** Adam I. Packard, Brian Lin, James E. Schwob

**Affiliations:** 1 Graduate Program in Cellular, Molecular and Developmental Biology, Sackler School of Graduate Biomedical Sciences, Tufts University, Boston, Massachusetts, United States of America; 2 Department of Developmental, Molecular and Chemical Biology, Tufts University, School of Medicine, Boston, Massachusetts, United States of America; Monell Chemical Senses Center, UNITED STATES

## Abstract

In the adult olfactory epithelium, the transcription factors Pax6 and Sox2 are co-expressed in sustentacular cells, horizontal basal cells (HBCs), and less-differentiated globose basal cells (GBCs)–both multipotent and transit amplifying categories—but are absent from immediate neuronal precursor GBCs and olfactory sensory neurons (OSNs). We used retroviral-vector transduction to over-express Pax6 and Sox2 individually and together during post-lesion recovery to determine how they regulate neuronal differentiation. Both Pax6 and Sox2, separately and together, can suppress the production of OSNs, as fewer clones contain neurons than with empty vector (EV), although this effect is not absolute. In this regard, Pax6 has the strongest effect when acting alone. In clones where neurons form, Pax6 reduces neuron numbers by comparison with EV, while Sox2 expands their numbers. Co-transduction with Pax6 and Sox2 produces an intermediate result. The increased production of OSNs driven by Sox2 is due to the expansion of neuronal progenitors, since proliferation and the numbers of Ascl1, Neurog1, and NeuroD1-expressing GBCs are increased. Conversely, Pax6 seems to accelerate neuronal differentiation, since Ascl1 labeling is reduced, while Neurog1- and NeuroD1-labeled GBCs are enriched. As a complement to the over-expression experiments, elimination of Sox2 in spared cells of floxed *Sox2* mice, by retroviral Cre or by *K5*-driven CreER^T2^, reduces the production of OSNs and non-neuronal cells during OE regeneration. These data suggest that Pax6 and Sox2 have counteracting roles in regulating neurogenesis, in which Pax6 accelerates neuronal production, while Sox2 retards it and expands the pool of neuronal progenitors.

## Introduction

Exposure of the olfactory epithelium (OE) to the outside environment puts the olfactory sensory neurons (OSNs) at risk of damage and death, which requires ongoing neurogenesis to maintain/restore sensory function [1]. Lifelong robust and regulated olfactory neurogenesis depends on two populations of neurocompetent olfactory stem cells: the horizontal basal cells (HBCs) and a subset of globose basal cells (GBCs) that are multipotent [2–6]. A cascade of transcription factors (TFs) downstream of the multipotent stem cell populations are known to be integral to neurogenesis, including Ascl1, Neurog1, and NeuroD1, listed in temporal order of their expression during embryogenesis and epithelial reconstitution [4,7–10]. However, the genetic regulation of the upstream multipotent progenitors is incompletely understood.

The TFs Sox2 and Pax6 are normally expressed in the adult OE within both GBC and HBC populations of multipotent progenitors, but they are absent from the population of immediate neuronal precursor GBCs (i.e., Neurog1- and NeuroD1-expressing) and from OSNs [4]. Following epithelial injury produced by inhalation of the olfactotoxic gas methyl bromide (MeBr), the spared GBCs and HBCs, which are jointly responsible for epithelial regeneration, express both TFs. However, as GBCs transition into immediate neuronal precursors and give rise to the OSNs directly, Sox2 and Pax6 become excluded from those populations [4].

Both Sox2 –an Sry, HMG-containing TF—and Pax6 –a paired box-containing TF—are candidate factors for regulating neurogenesis in other neuronal tissues. In the retina, Pax6 preserves progenitor cell multipotency, while in the subventricular zone Pax6 dosage controls the transition from type C transit amplifying cells to type A neuroblasts [11–13]. Moreover, Pax6 is critical for the initial development of the OE [14,15] and apparently for its recovery after injury [16]. Sox2 is essential for somatic cell reprogramming and for maintaining neuronal stem cells in an undifferentiated state [17–21]. Its dosage is also critical in regulating neurogenesis, influencing whether neural stem cells progress toward neuronal differentiation or not [22–24]

In addition to the exclusion of Sox2 and Pax6 from immediate neuronal precursors and neurons of the developing, normal, and regenerating OE, Sox2 is universally expressed throughout the OE of *Ascl1* mutants, in which progenitors are incapable of neuronal commitment [4,25]. These expression patterns, along with their established roles in suppressing neural differentiation among other neuronal tissues, suggest that Sox2 and Pax6 also hold olfactory progenitors in an undifferentiated state. To test this hypothesis we over-expressed each factor individually and both of them together via retroviral transduction. We also accomplished genetic deletion of Sox2 via retroviral transduction and conditional deletion via Cre recombinase. We find that Pax6 and Sox2 have complex effects on the function of transduced progenitor cells. Both TFs when transduced either individually or together reduce the number of OSN-containing clones. However, the Sox2-transduced clones that escape suppression contain a greatly expanded population of neurons, while Pax6 transduction reduces their number by comparison with EV; when expressed together the effects balance out.

## Materials and Methods

### Constructs

**Fig 1:**
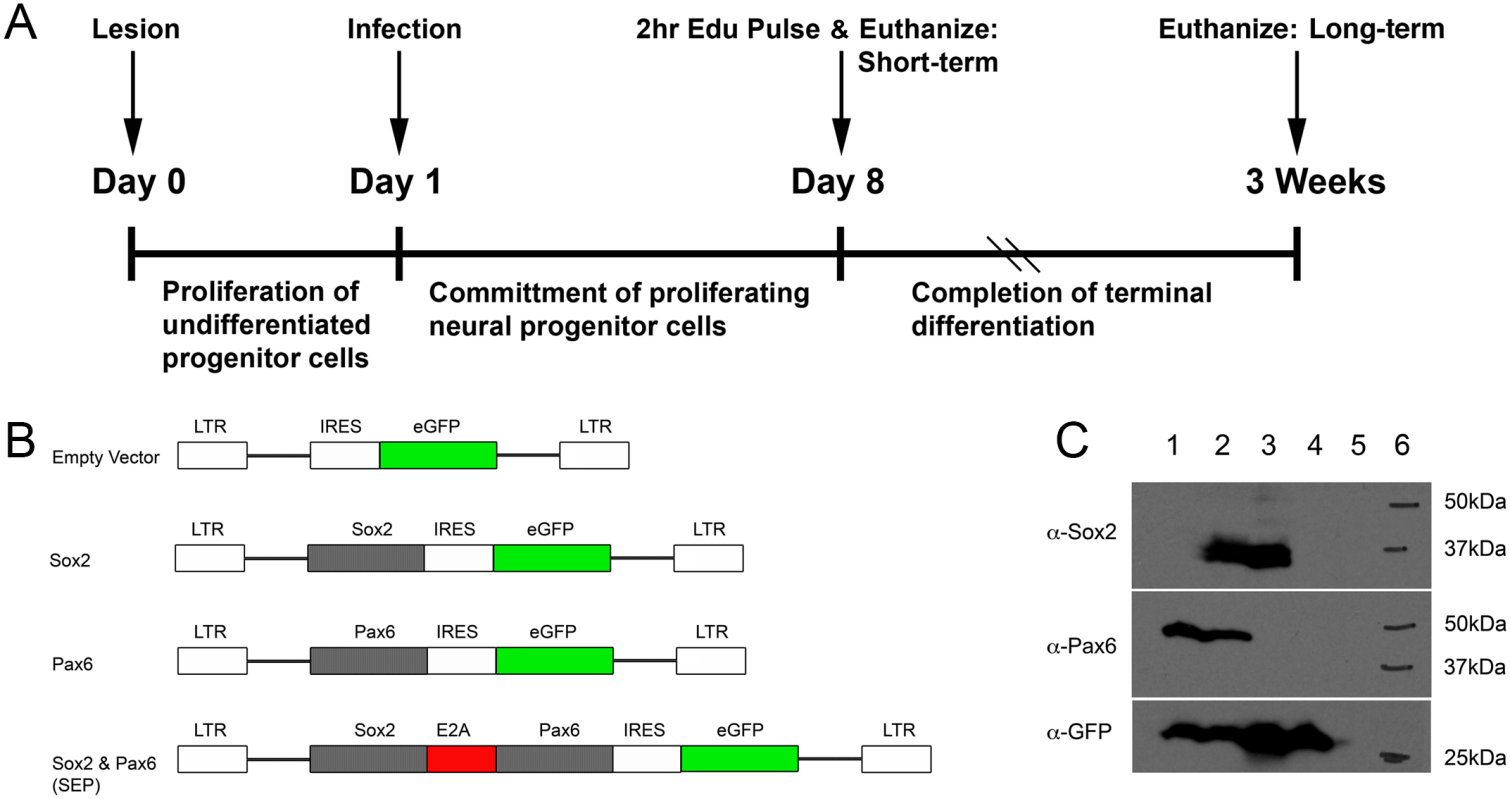
Experimental paradigm and construct design. (A) Timeline of experimental manipulations used in these studies. (B) Map outlining the genetic maps of the various retroviral vectors used in these studies. Each viral vector has the coding sequence of one or two genes in its multiple cloning site, which sits 5’ (upstream) of an IRES-eGFP sequence. The vectors are identified in name by their respective inserts: empty vector, *Sox2*, *Pax6*, and *SEP* (*Sox2-E2A-Pax6*). (C) Western blot performed on lysates from HEK293T cells transfected with the vectors, confirming protein production of each gene product at the expected molecular weights when stained with antibodies against Pax6, Sox2 and GFP. Lane 1: pLIA-Pax6, lane 2: pLIA-SEP, lane 3: pLIA-Sox2, lane 4: pLIA-GFP, lane 5: untransfected HEK cells, lane 6: molecular weight standards.

All viral vectors are modified from *pLIA-IRES-eGFP*, a MoMuLV-, pBabe-derived retrovirus (see Fig 1) [26]. The coding sequence for *Sox2*, *Pax6*, *Cre*, or *Sox2-E2A-Pax6* (*SEP*) were cloned upstream of the *IRES-eGFP* in the vector’s multiple cloning site. To generate the *SEP* sequence, homologous overlapping arms corresponding to the E2A peptide sequence from the equine rhinitis-A virus were PCR cloned onto the 3’ end of *Sox2* and 5’ end of *Pax6*, respectively, [27], and then a further round of PCR was used to create a fusion construct that consists of a single cistron and leads to bands of near-equal intensity of expression of Sox2 and Pax6 via ribosomal skipping, as shown in Western blots (see Fig 1) [28,29]. By way of contrast, the use of an IRES sequence between Sox2 and Pax6 resulted in a substantial diminution of the expression as measured by Western Blot relative to the use of the E2A sequence in the construct (data not shown). Primers for SEP are as follows: SoxE2A_R: 5’- TTTCAACATCGCCAGCGAGTTTCAACAAAGCGTAGTTAGTACATTGCCCACTACCCA TGTGCGACAGGGGC, Pax6E2A_F: 5’- CTTTGTTGAAACTCGCTGGCGATGTTGAAAGTAACCCCGGTCCTATGCAGAACAGTCACA GC, BamSox_F: 5’- CGCGGATCCCGATGTA TAACATGATGGAGACGGAGCTGAAG, PaxXho_R: 5’- CGCTCGAGCGGTTACTGTAAT CGAGGCCAGTAC. All viral vectors used in these studies express *eGFP* downstream of an IRES sequence in order to trace infected cells and their progeny.

### Viral production

Viral particles were assembled by transiently transfecting *ϕnx* cells with purified retroviral plasmids. *ϕnx* cells stably expressing the *gag*, *pol*, *env* genes provide ecotropic packaging of the viral genome, after which particles shed in the medium were collected and concentrated by dialysis [30]. Viral concentrate, with titers ranging from 5 x 10^6^ to 5 x 10^7^ pfu, was stored at -80°C until use.

### Animals

The generation of conditional *Sox2* knock-out mice, *Sox2*^*flox/flox*^, is described elsewhere [23]. They were generated with loxP sites spanning a 6.5kb fragment that incorporates the *Sox2* open reading frame (ORF) along with portions of 5’ and 3’ untranslated regions surrounding it, leading to complete deletion of the *Sox2* ORF, and its translated product, upon expression of *Cre recombinase*. The *Sox2*^*flox/flox*^ mice (the generous gift of S. Nicolis) were maintained on an FVB/N background. *Rosa26-loxP-stop-loxP-LacZ* (*R26*^*fl(stop)LacZ*^*)* indicator mice were used for lineage analysis and were purchased from the Jackson Laboratories (*B6*.*129S4-Gt(Rosa)26Sor*^*tm1Sor*^*/J*, stock #003474) [31]. *K5 promoter-CreER*^*T2*^ (*K5*^*CreERT2*^) transgenic mice have been described previously [6] and are used to selectively drive recombination in HBCs. The following genotypes were bred: bigenic *Sox2*^*flox/flox*^*;R26*^*fl(stop)LacZ*^ (SR) and trigenic *K5*^*CreERT2*^*; Sox2*^*flox/flox*^*; R26*^*fl(stop)LacZ*^ (KSR). Wild type adult C57/B6 mice were purchased from JAX (stock# 000664). Tail tissue harvested for genotyping was digested in 300μL of 50mM NaOH for one hour at 95°C, vortexed, and 16.7 microliters of 1M Tris-HCl pH 8 was added. The samples were vortexed and spun down at 13,000 x g for 6 minutes. 3 μL of the DNA prep was used in separate reactions to identify the appropriate amplicons for transgene-carrying pups. Genotyping primers for *Sox2*^*flox/flox*^, *R26*^*fl(stop)-LacZ*^, and *K5*^*CreERT2*^ are as follows: *Sox2*^*flox*^ forward 5’–GACCTAGCCAGACCCCCTTA, *Sox2*^*flox*^ reverse 5’–AGATAAGTGGGAGGTT AAGCGAGG, *Rosa26* WT: 5’–GGAGCGGGAGAAATGGATATG, *Rosa26*^*fx(stop)*^: 5’–GCGA AGAGTTTGTCCTCAACC, *Rosa26* common: 5’–AAAGTCGCTCTGAGTTGTTAT, *K5-CreER*^*T2*^: forward 5’–GTCATGAACTATATCCGTA ACCTGGATAG, reverse 5’–GCTGGGGCATGAAGGCGGTGGGCG.

All mice were maintained on *ad libitum* rodent chow and water. All animals were housed in a heat- and humidity-controlled, AALAC-accredited vivarium operating under a 12:12-hour light-dark cycle. All protocols describing the use of vertebrate animals were approved by the Committee for the Humane Use of Animals at Tufts University School of Medicine, where the animals were housed and the experiments were conducted.

### MeBr lesion

For purposes of *in vivo* retroviral transduction, 12-week old male mice were exposed to the olfactotoxic gas methyl bromide (MeBr) at 165 parts per million (ppm), mixed with air, for a duration of eight hours [2,32]. Because of strain differences in MeBr sensitivity, *R26*^*fl(stop)-LacZ*^ mice were exposed to 170 ppm MeBr, as were *Sox2*^*flox/flox*^, and bigenic SR mice.

### Viral infection

Mice were brought to a surgical anesthetic plane by intraperitoneal injection of a triple anesthetic cocktail of Ketamine (21.5 mg/kg), Xylazine (4.3 mg/kg) and Acepromazine (0.7 mg/kg), then tracheotomized for airway patency and subjected to palatal occlusion to contain the intransal infusion of the virus. 70 μl of viral concentrate was infused intranasally, along with 40 μg/ml of polybrene, by threading (PE-10) polyethylene tubing 7mm into the left naris. Mice were left anesthetized and tracheotomized for 3.5 hours to facilitate infection. Wild type adult C57/B6 mice were used for infection with EV, Pax6, Sox2 and SEP, while monogenic *Sox2*^*flox/flox*^, *R26*^*fl(stop)-LacZ*^ and bigenic SR mice were used for Cre infections. Animals were infected 1-day after exposure to MeBr and maintained for either 8 days or 3 weeks following infection, at which point they were euthanized for histological processing and evaluation; see Fig 1 for the timeline of experiments.

### HBC-specific recombination

KSR mice were injected intraperitoneally with tamoxifen at 200 mg/kg suspended in 150 μl of corn oil to generate homozygous Sox2-mutant HBCs. The treated mice were exposed to MeBr 2 weeks later, which results in activation to multipotency of a large percentage of HBCs and in the derivation of GBCs, neurons, and non-neuronal cells directly or indirectly from the activated HBCs. The composition of clones that derive from the activated, mutated HBCs was assessed using immunohistochemistry with cell-type specific reagents (see below).

### EdU injection

Mitotically cycling epithelial cells were labeled by the incorporation of 5-ethynyl-2'-deoxyuridine (EdU; A10044, Invitrogen). Mice were injected intraperitoneally (IP) with 50 mg/kg EdU at a concentration of 10 mg/ml in PBS. After a 2-hour chase period, the mice were perfused with fixative, dissected and processed. Visualization is described below.

### Tissue processing

RVV-infected mice, after the appropriate survival times, were deeply anesthetized by injection with a cocktail of Ketamine (37.5 mg/kg), Xylazine (7.5 mg/kg) and Acepromazine (1.25 mg/kg), transcardially flushed with PBS, and then perfused with one of a variety of fixatives that depended on the purpose of the experiment. The fixatives included 4% paraformaldehyde (Fisher Scientific, Suwanee, GA) in 0.05 M sodium phosphate buffer, pH 7.2 (for immunochemistry) and 1% paraformaldehyde/0.1% glutaraldehyde (for X-gal staining), with 2–4 hours of post-perfusion fixation under vacuum. Tissues were rinsed with PBS, cryoprotected by immersion in 30% sucrose in PBS and then frozen in OCT compound (Miles Inc.,Elkhart, IN). The olfactory mucosa was sectioned on a Leica cryostat in the coronal plane; 8μm sections were collected on to "Plus" slides (Fisher Scientific) and stored at -20°C for future applications.

### Immunohistochemistry & Histochemistry

**Fig 2:**
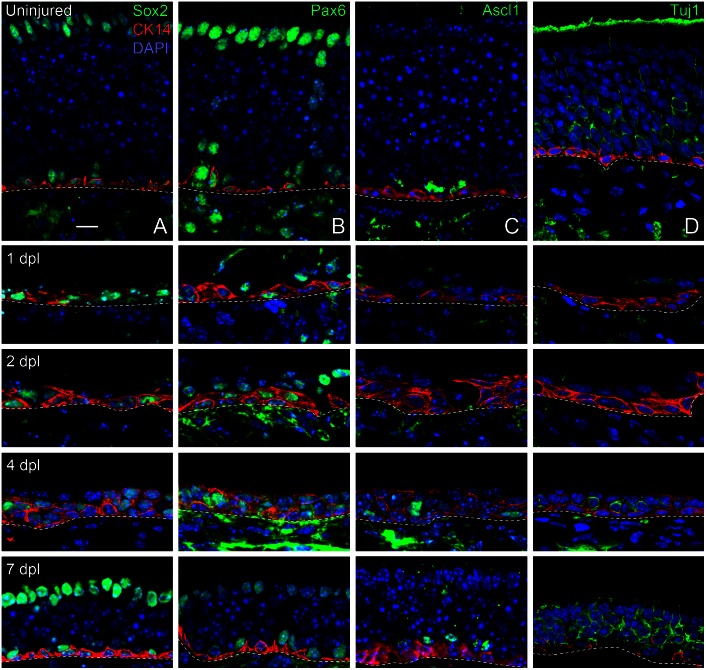
Sox2, Pax6, and Ascl1 demonstrate the hierarchy of progenitor cell progression during MeBr-induced regeneration in mouse olfactory epithelium (OE). (Col. A) Sox2, (Col. B) Pax6, (Col. C) Ascl1 (Mash1), and (Col. D) TuJ1 (neuron-specific tubulin) immunostaining in control mouse OE and at 1, 2, 4 and 7 days post MeBr-lesion (dpl). Note the reappearance and patterning of transcription factor expression. CK14 labeling marks the horizontal basal cells in the uninjured epithelium and the cells that derive from them at the early stages in the reconstitution of the epithelium, including ones that transition into Sox2/Pax6 (+)/CK14 (-) GBCs. Regeneration of olfactory sensory neurons is marked by the expression of neuron-specific tubulin (marked by Tuj1 staining). The vast majority of spared cells express both Pax6 and Sox2 up through 4 dpl. By this time Ascl1-expressing GBCs have made a limited reappearance and a few Tuj1 (+), neurons have emerged. Dashed lines mark the basal lamina. Scale bar in (A, uninjured) equals 10 μm and applies to all panels.

**Fig 3:**
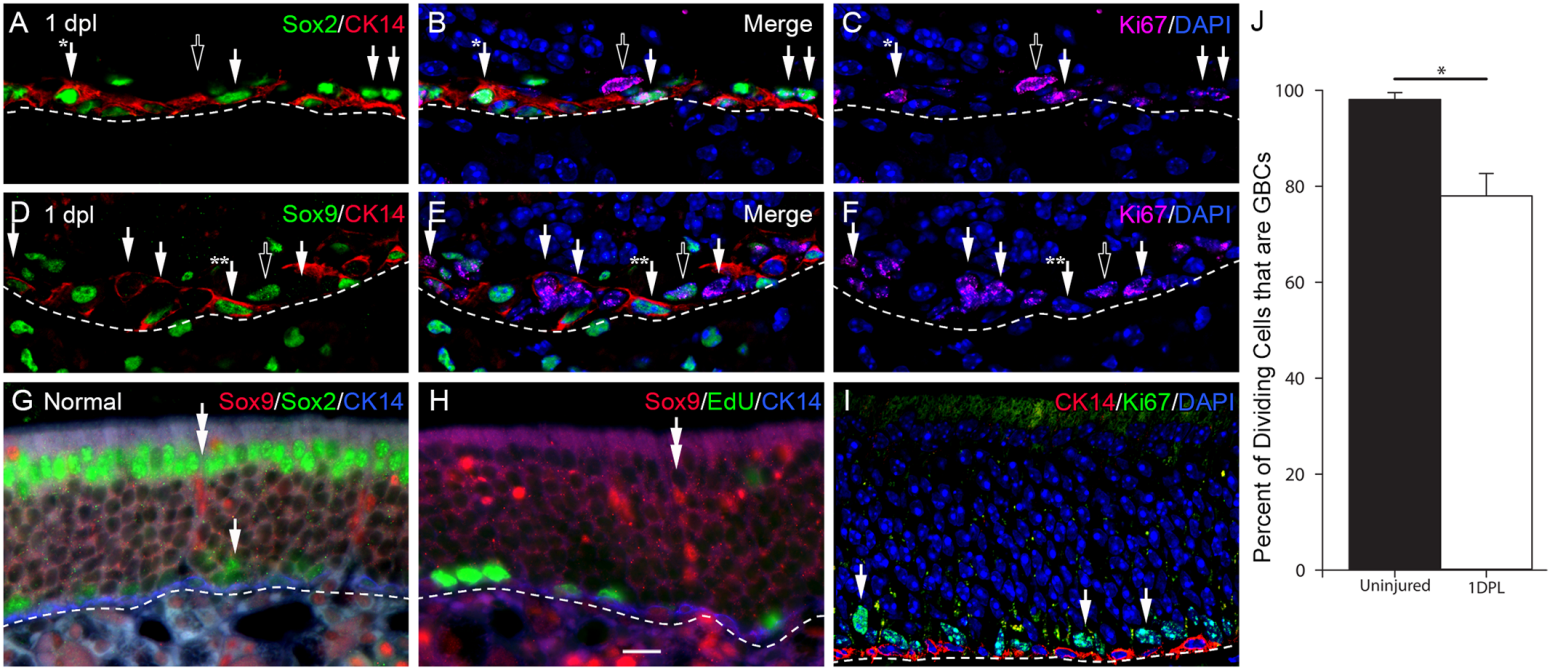
Identification of cellular targets for replication-incompetent retroviruses, during lesion-induced regeneration of the OE. (A-F) At 1 day post-MeBr lesion GBCs and duct/gland cells are predominant proliferating populations and putative retroviral targets as shown by immunostaining of tissue sections of OE from 1 day post-MeBr lesion with antibodies to Sox2 (which mark GBCs) and to Sox9 (which marks gland/duct cells) along with anti-CK14 and Ki-67, the latter marking proliferating cells. Single solid arrows mark Ki-67 (+), dividing cells that are Sox2 (+)/CK14 (-) in A-C and Sox9 (-)/CK14 (-) in D-F, and are found at a remove superficial to the basal lamina and correspond to GBCs or HBCs caught in transition to GBCs. All of the CK14 (+) cells are also Sox2 (+). In A-C, the solid arrow with one asterisk indicates a dividing HBC, while in D-F the solid arrow with two asterisks marks an HBC that is not dividing but now expresses Sox9, in addition to the Sox2 expression documented above. The presence of Sox2 (+)/Sox9 (+) basal cells may represent HBCs that are differentiating into duct/gland cells, which is observed after MeBr lesion (Leung et al., 2007). In A-F the hollow arrows indicate a proliferating putative duct/gland cell (in D-C) and a marker-confirmed duct/gland cell (in D-F). (G-I) Staining of sections of the normal mouse olfactory epithelium with Sox2, Sox9, and CK14 to mark specific cell types along with markers of proliferation, either Ki-67 labeling or EdU incorporation, for comparison with the composition of the epithelium after injury. On the basis of these staining patterns, Sox2 (+) cells located at a distance superficial to the basal lamina with weak or no CK14 (+) labeling are classified as GBCs, while those basal cells that are attached to the basal lamina and strongly positive for CK14 are identified as HBCs. Duct/gland cells or cells that are Sox9 (+) form a separate category. (J) The criteria enumerated above were used to count the percent of dividing cells that are GBCs, which are the vast majority under the lesion conditions used here. Dashed lines indicate the basal lamina. Scale bar in K corresponds to 10 μm and applies to all photomicrographs.

**Fig 4:**
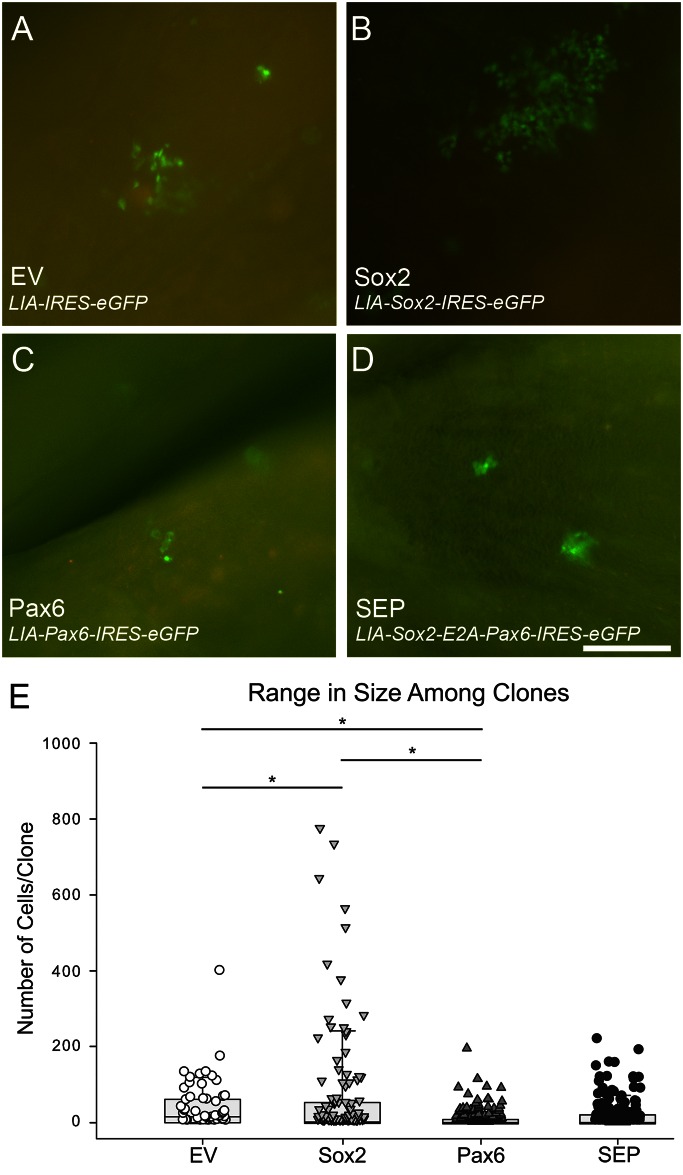
*Sox2* transduction produces the largest clones while *Pax6* produces the smallest. (A-D) GFP expression in representative whole mounts, demonstrating typical clone size following transduction with *EV*, *Sox2*, *Pax6*, and *SEP* retroviral vectors, respectively. Scale bar in (D) corresponds to 50 μm and applies to all photos. (E) The range in cell number per clone is depicted as a box-and-whiskers scatter plot, and the median clone size is indicated by the horizontal black line within the box. The number of clones analyzed were as follows: *EV*– 49, *Sox2*–111, *Pax6*–150, and *SEP*– 165. Non-parametric ANOVA was statistically significant (Kruskal-Wallis, *p <* 0.05) as were post-hoc pairwise comparisons using Conover-Iman tests of multiple comparisons using rank sums; * designates p < 0.05.

**Fig 5:**
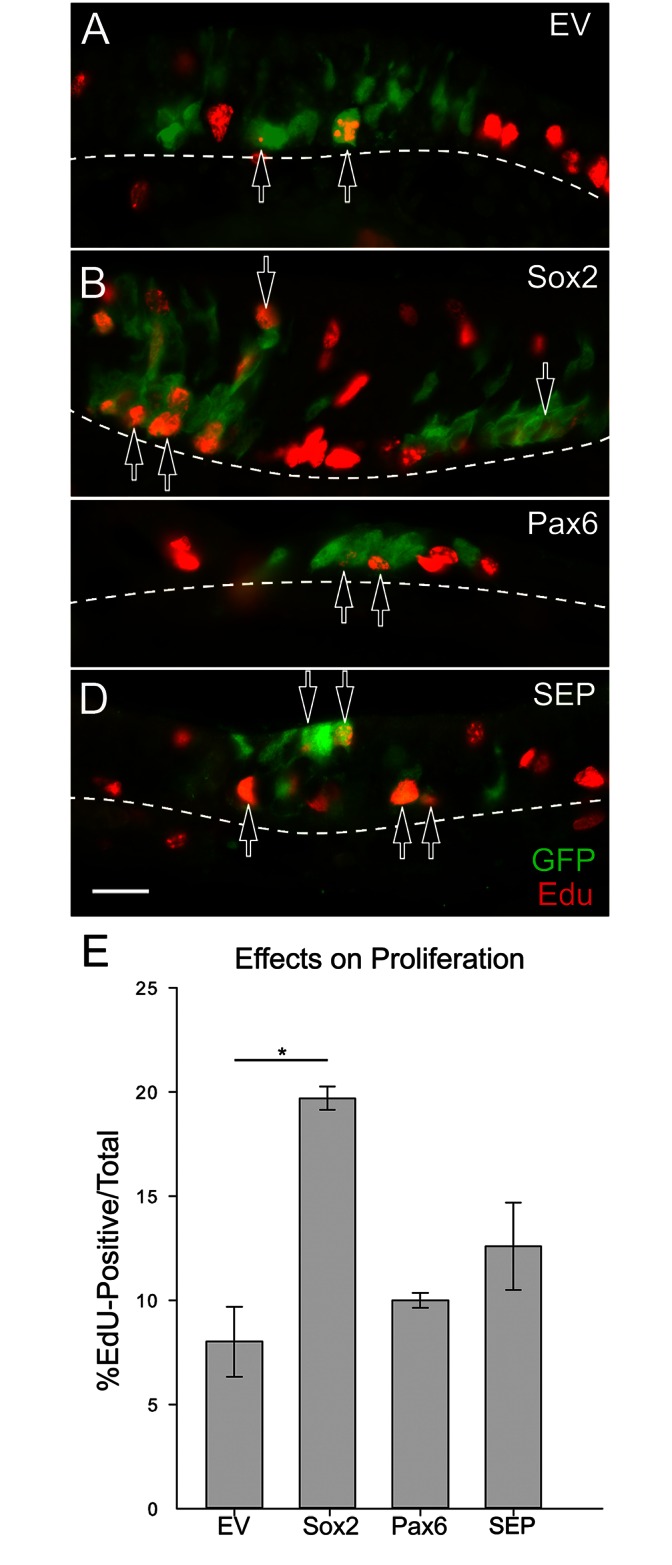
*Sox2* transduction increases the number of cells proliferating early on in the regeneration process, compared to control. (A-D) Proliferation of transduced cells was assessed, across viral constructs at 8 days post-infection by labeling with GFP and EdU (to identify cells in S-phase at the time of euthanasia). Hollow arrows indicate examples of GFP (+)/EdU (+) cells. Note the large number of double-labeled cells with Sox2 transduction (B). Dashed lines mark the basal lamina, and the scale bar in (D) corresponds to 20 μm and applies to all of the images. (E) The mean number of EdU (+) cells is plotted as a percentage of the total number of GFP (+) cells (percentages were derived from count data justifying arcsin-transformation to allow for multiple testing by Kruskal-Wallis ANOVA, with Dunn’s method for pairwise comparisons, * p < 0.05).

**Fig 6:**
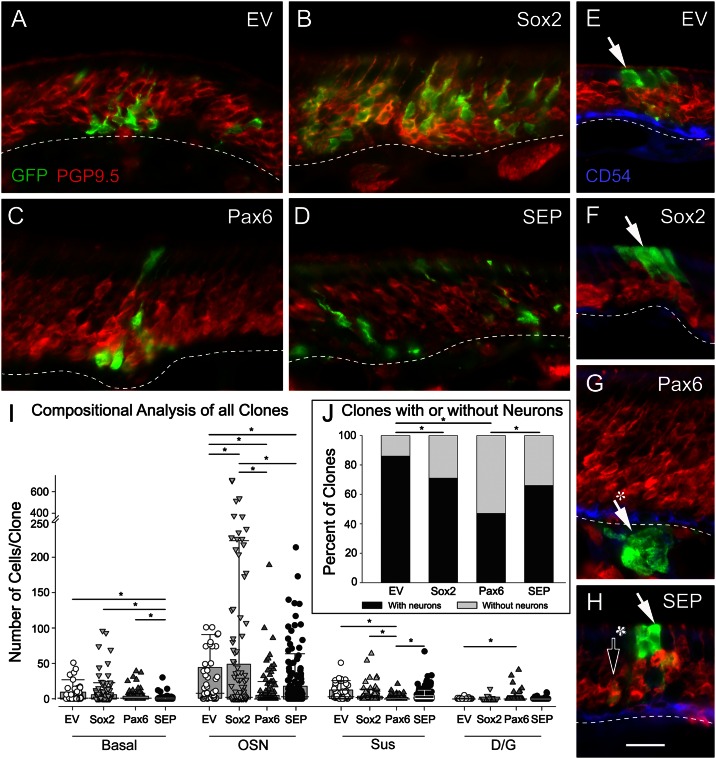
Pax6- and Sox2-encoding retroviral vectors differentially alter the cellular composition across clones. (A-H) Tissues harvested 3 weeks after *EV*, *Pax6*, *SEP* and *Sox2* RVV-transduction were stained with GFP, along with PGP9.5 (a neuronal marker) to illustrate the range in outcomes for each condition. Cell identity among clones was then determined relative to PGP9.5 staining. GFP-labeled cells were identified as sustentacular cells (Sus) when their cell bodies were found superficial to the band of PGP9.5 (+) OSNs, while GFP-labeled cells were identified as basal cells if they were deep to the band of neurons. GFP (+) cells were identified as duct/gland cells (D/G) when they were flattened, oriented as a chain of cells along the apical-basal axis, and/or extended deep to the basal lamina into the lamina propria (cf. Fig 3G and 3H). GFP (+) cells were identified as OSNs, if they co-labeled with PGP9.5. (E-H) CD54 was used to immunostain HBCs along with PGP9.5. (E-H) There is substantial variation in clonal composition. (E) The arrow indicates a mixed clone composed of OSNs and a large group of Sus cells atop the neurons. (F) The arrow indicates a Sus-only clone where the GFP-labeled cells sit atop the neurons. (G) The solid arrow with asterisk points to gland cells located deep to the HBCs and basal lamina. (H) Another example of a mixed clone composed of Sus cells (arrow) and neurons (hollow arrow with asterisk). Dashed lines mark the basal lamina (A-H) and the scale bar in (H) corresponds to 20 μm and applies to all of the panels. (I) For the various cell types, the data from each and every clone was used to generate a box-and-whiskers scatter plot; the median for each distribution is marked by the horizontal black line within the box. In the order shown in the graph they are: for basal cells– 1, 1, 1, 0; for OSNs– 8, 2, 0, 3; for Sus cells– 3, 2, 0, 1; for D/G (duct/gland cells)– 0, 0, 0, 0, respectively. Note the break in the ordinate of the graph to accommodate those clones that contain a markedly greater number of neurons, which occur exclusively with Sox2 transduction. Because all of the datasets shown here failed the Shapiro-Wilk test for normality, a Kruskal-Wallis One-Way ANOVA on Ranks was used, followed by Dunn’s Method for pairwise multiple comparisons. (J) For the group of clones as a whole, the percentage of clones that contain neurons for each form of transduction are indicated in the bar graph, and multiple pairwise comparisons (indicated by the horizontal lines) are significantly different (Fisher Exact test with Holm-Sidak multiple comparison correction, * p < 0.05).

**Fig 7:**
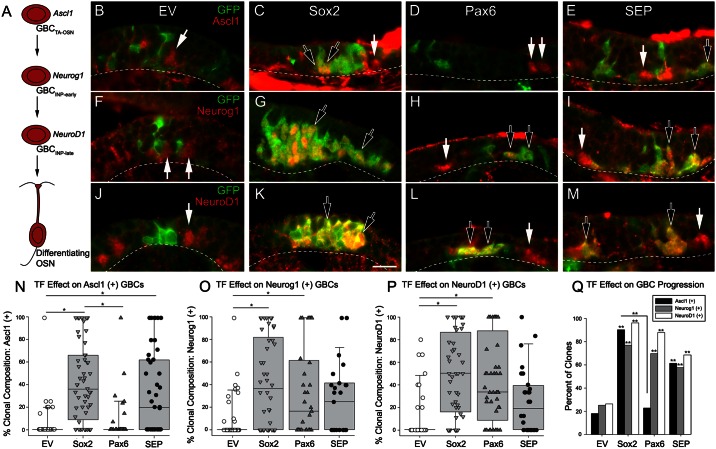
*Sox2* transduction enhances Ascl1 expression, whereas *Pax6* transduction leads to a reduction in Ascl1 expression and a reciprocal increase in the prevalence of downstream neural precursor markers. (A) A proneural bHLH cascade operates during olfactory neurogenesis and defines a hierarchy of GBCs as they progress from transit amplifying (TA) progenitor cells into differentiated neurons, by way of two immediate neural precursor (INP) stages. (B-M) At 8-days post-infection (9-days post-lesion), tissue was immunolabeled for Ascl1, Neurog1, and NeuroD1 along with GFP in clones derived from *EV* (B, F, J), *Sox2* (C, G, K), *Pax6* (D, H, L) and *SEP* (E, I, M) RVV-transduction. Hollow arrows mark GFP (+) cells within a given clone that co-express Ascl1, Neurog1, or NeuroD1. In contrast, solid arrows mark non-transduced, i.e., GFP-lacking, cells that express these factors. Dashed lines mark the basal lamina and scale bar in K corresponds to 20 μm in panels B-M. (N-P) Quantitative analysis of the effect of the various transduction conditions on the percentage of cells in a clone that express Ascl1 (N), Neurog1 (O) and NeuroD1 (P). For the graph, the percentage of cells marked by joint expression of the individual transcription factor and GFP within each clone are graphed in a box-and-whiskers scatter plot; the median number of each distribution is marked by the horizontal black line within the box. For Ascl1+ GBCs, the median percentages for EV vs. Sox2 vs. Pax6 vs. SEP are 0, 36, 0, 20, respectively. For Neurog1+ GBCs, the median percentages were 0, 37, 17, 25, respectively. Finally, for NeuroD1+ GBCs, the median values were 0, 50, 33, 19, respectively. Multiple pair-wise comparisons (horizontal line) were found to be statistically significant using Dunn’s method (* p < 0.05) following the demonstration of overall significance using Kruskal-Wallis One-Way ANOVA tests. (Q) Percentage of clones that contain cells positive for labeling with the bHLH factors. To compare percentages statistically, data were first arcsin transformed prior to normality testing, standard parametric ANOVA, and post-hoc Holm-Sidak pair-wise testing (**p < 0.01). Asterisks signify comparisons to EV, except for the comparison in number of Ascl1-conatining clones between Sox2 and Pax6 vectors, which is designated by the orthogonal lines.

**Fig 8:**
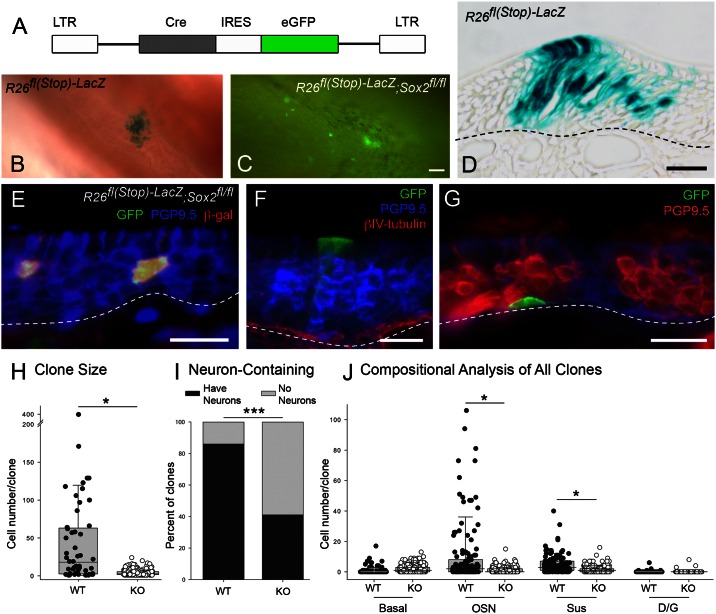
Conditional deletion of *Sox2* by way of RVV transduction produces smaller clones that contain fewer neurons than control. (A) Cartoon illustration of retroviral construct used for transduction with *Cre* recombinase. (B, C) Representative clones from whole mount tissue harvested 3 weeks after infection. (B) X-gal staining demonstrates the typical clone size from control *R26*^*fl(stop)-LacZ*^ indicator mice transduced with *Cre*. (C) GFP fluorescence expressed from the RRV, demonstrates the typical clone size from *Sox2*^*flox/flox*^ transduced mice. (D) Representative histological section stained to detect β-galactosidase activity within a clone following *Cre*-transduction in a *R26*^*fl(stop)-LacZ*^ control mouse; note the large numbers of neurons, which are situated broadly along the middle of the epithelium. (E-G) Representative sections from *Cre/GFP*-expressing clones in *Sox2*^*flox/flox*^ mice that have been immunostained to characterize clonal composition. Dashed lines mark the basal lamina. (H) The range in the number of cells/clone is depicted in the form of a box-and-whiskers scatter plot, and the horizontal black line within the box indicates the median clone size, where the median values were 19 and 3, respectively. The distributions are significantly different (Mann-Whitney-U test, *** *p* < 0.001). (I) The proportion of infected clones containing neurons or not is depicted in the form of a stacked bar graph, gray and black respectively. The proportions are significantly different between wildtype and knockout (Z test, *** p < 0.001) (J) Compositional analysis plotting the number of the different types of cells in each clone, by a box-and-whiskers scatter plot; median clone size is indicated with a black line. Median values for control vs. knockout for basal, OSN, Sus, and D/G are 0 vs. 1, 2 vs. 0, 3 vs. 1, and 0 vs. 0, respectively. Note that both the number of neurons and the number of Sus cells are significantly reduced by Cre-mediated recombination in floxed *Sox2* mice as shown by Dunn’s method for pairwise comparisons (indicated by the horizontal lines, * p < 0.05). Scale bar in (C) corresponds to 50 μm and also applies to B, scale bars in D-G correspond to 10 μm.

**Fig 9:**
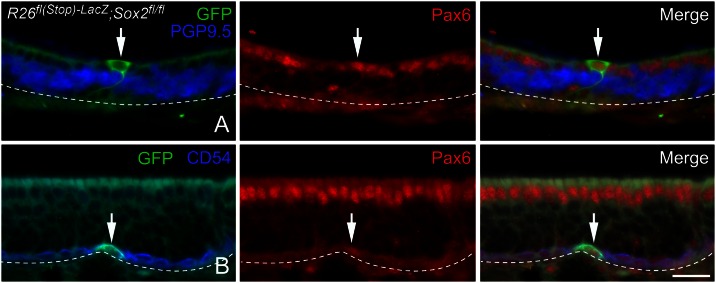
Pax6 expression remains unaltered within Sus cells and HBCs upon conditional deletion of *Sox2*. Clones from tissue harvested 3-weeks after *Cre* RVV-transduction of bigenic *R26*^*fl(stop)-LacZ*^*; Sox2*^*flox/flox*^ mice were immunolabeled for the neuronal marker PGP9.5 (A) or the HBC marker CD54 (B), along with Pax6 and GFP. In clones containing GFP (+)/ PGP9.5 (-) Sus cells (A), situated above the neural strata (arrows), Pax6 expression remains indistinguishable from surrounding untransduced Sus cells. In clones containing GFP (+)/CD54 (+) HBCs (B), Pax6 levels remain indistinguishable from untransduced HBCs (arrows). Arrowheads mark the basal lamina and scale bar corresponds to 20 μm and applies to all panels.

**Fig 10:**
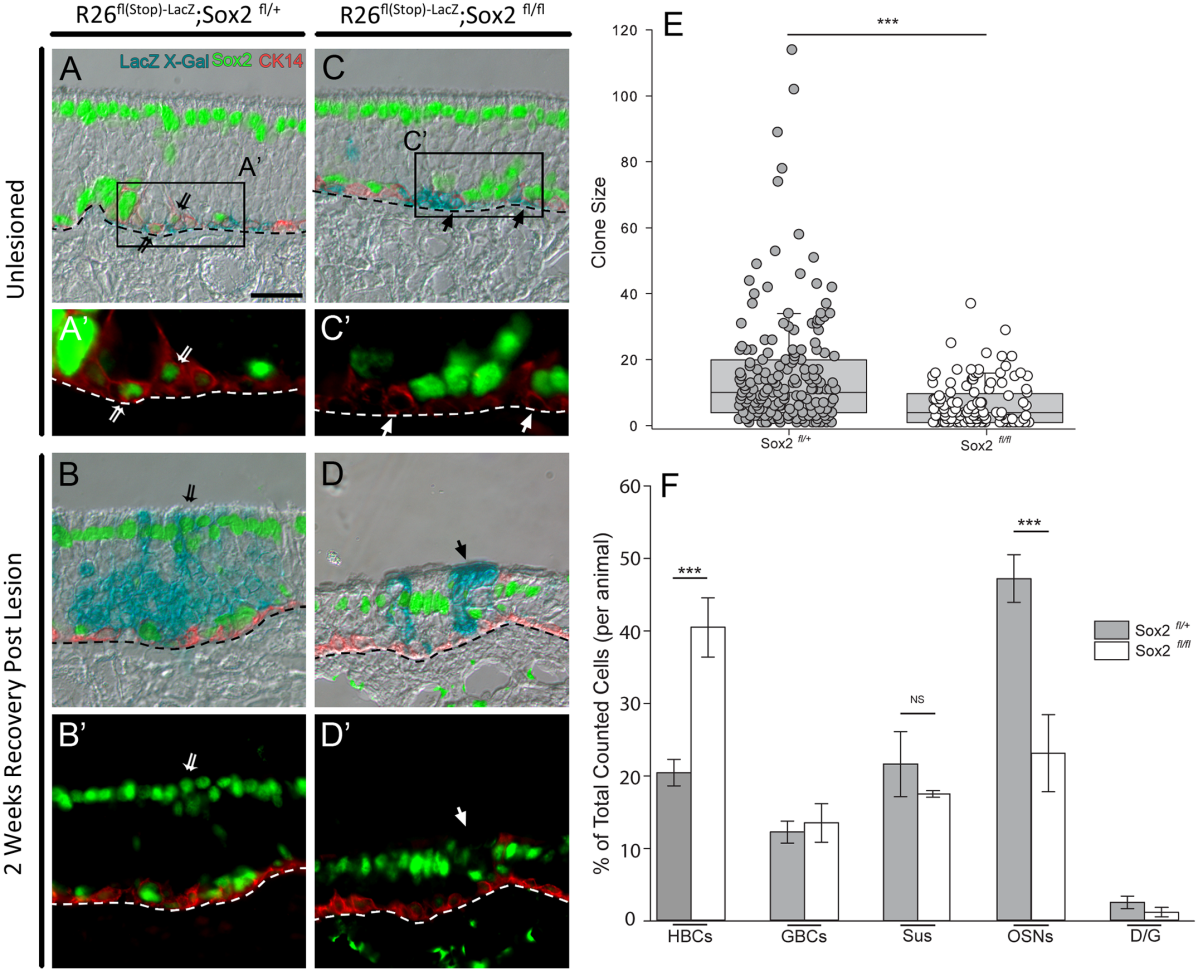
CreER^T2^-mediated excision of *Sox2* in HBCs reduces clone size and neuron numbers. Tamoxifen was administered to unlesioned (A) and lesioned (B) control *K5*^*CreERT2*^*; Sox2*^*flox/+*^*; R26*^*fl(stop)-LacZ*^ mice, as well as to unlesioned (C) and lesioned (D) experimental *K5*^*CreERT2*^*; Sox2*^*flox/flox*^*; R26*^*fl(stop)-LacZ*^ mice; tissue was analyzed three weeks into epithelial recovery. Sections are X-gal-stained to detect β-galactosidase activity within clones, stained for cytokeratin 14 to mark HBCs, and immunolabeled for Sox2 to confirm gene recombination. (A) In the mice that are heterozygous for the floxed *Sox2* allele, the excision of one copy of *Sox2* had no discernable effect in the absence of lesion. (A’) The inset shows Sox2 labeling in β-galactosidase-marked CK14 (+) basal cells, as expected (double thin arrows). (B, B’) In the heterozygous animals, excision of one copy of *Sox2* had no effect on the composition of the clones that arise following MeBr-lesion from the activated HBCs. X-gal-labeled Sus cells maintain their prominent expression of Sox2 following their regeneration from the HBCs that have undergone genetic recombination, as expected (double thin arrows). (C) In the unlesioned, homozygous floxed *Sox2* mice, the absence of Sox2 has no apparent effect. (C’) The inset shows the absence of Sox2 labeling in β-galactosidase-marked CK14 (+) basal cells, as expected (single arrows). (D, D’) However, when the Tamoxifen-treated, homozygous floxed-*Sox2* mice were exposed to MeBr, the X-gal-stained clones resulting from lesion-induced activation of the HBCs were smaller and contained fewer neurons. X-gal-labeled Sus cells lack their usual prominent expression of Sox2 following their regeneration from the HBCs that have undergone genetic recombination, as expected (single arrows). (E) Comparing lesioned tissue between floxed *Sox2* homozygotes and heterozygotes, the range in the number of cells/clone is depicted in the form of a box-and-whiskers scatter plot, and the median clone size is indicated by the horizontal black line within the box. The median value of clone size between heterozygote vs. homozygote is 10 vs. 4, respectively. The difference between homozygotes and heterozygotes is statistically significant (Mann-Whitney U test, *** p < 0.001). (F) Compositional analysis plotting the relative number of different cell types in each clone, reveals that the percentage of neurons (OSNs) is significantly decreased in the floxed *Sox2* homozygotes (white bars) compared to heterozygotes (black bars) (Mann-Whitney U-test, *** p < 0.001), while the percentage of HBCs is increased (Mann-Whitney U-test, *** p < 0.001). Scale bar in A is 25 μm and applies to all panels except A’ and C’. The dashed lines mark the basal lamina.

Standard laboratory protocols were used to detect the expression pattern of individual proteins in normal OE and RVV-infected OE (Table 1 and Figs 2–10) [4,33]. Adequate labeling with a number of the antibodies required a series of pretreatments to the sections prior to antibody incubation. Briefly, frozen sections were rinsed in PBS for 5 minutes to remove the OCT, puddled with 0.01 M citric acid buffer (pH 6.0), and then placed in a commercial food steamer containing water in its reservoir heated to 90°C for 15 minutes (which we term “steaming”). After cooling, sections were rinsed with PBS briefly before incubating with blocking solution (10% serum + 5% Non fat dry milk + 4% BSA + 0.01% Triton X-100) for 30 minutes at room temperature. The analyses conducted here depended on a number of double- and triple-immunohistochemical staining approaches (Table 2). In all cases, the sections were incubated with primary antibodies overnight at 4°C. Bound primary antibodies were visualized either by incubation with the corresponding biotinylated secondary antibody followed by avidin-biotinylated HRP conjugate (Elite ABC Kit, Vector Laboratory, Burlingame, CA) and then 3, 3'-diaminobenzidine (DAB) as chromogen, or with one of several fluorescently conjugated secondary antibodies for purposes of co-localization by double and/or triple labeling. On occasion, tyramide signal amplification (TSA) was utilized to enhance a weak signal or permit staining with two antibodies from the same species, and used according to kit instructions (PerkinElmer, Waltham, MA). For detection of *LacZ*, tissue was reacted with 5- bromo-4-chloro-3-indolyl-d-D-galactopyranoside solution (X-gal, Sigma cat# 16555) according to previously published procedures (Figs 8 and 10) [5].

**Table 1:** Antibodies used in the study.

Primary antibody	Source and catalog number	Immunogen and preparation
Mouseα βIII-Tubulin (TuJ-1)	Covance, cat. no. MMS-435P	Microtubules derived from rat brain.
Goatα βGal	Biogenesis, cat. no. 4600–1409	Bacterial βGalactosidase, 119 kDa full length protein
Goatα Sox2	Santa Cruz, cat. no. sc-17320	Peptide sequence near C-terminus, amino acids 277–293 of human Sox2 (YLPGAEVPEPAAPSRLH), affinity purified serum
Rabbitα Sox2	ProteinTech, cat. no. 11-64-1-AP	Recombinant full-length human Sox2
Mouseα Pax6	Developmental Studies Hybridoma Bank	Recombinant protein corresponding to N-terminus of chicken Pax6, amino acids 1–223
Rabbitα PGP9.5 (UCHL1)	Cedarlane/Ultraclone, cat. no.31A3	Purified human PGP9.5 protein (ubiquitin-C terminal hydrolase 1, 27 kD) from brain
Mouseα Ascl1 (Mash1)	BD-Pharmingen, cat. no. 556604 (clone 24B72D11.1; Lo et al, 1991)	Recombinant-full length rat MASH1 protein, affinity chromatography-purified
Goatα Neurog1	Santa Cruz, cat. no. sc-19231	Peptide sequence near N-terminus of mouse Neurog1 (ARLQPLASTSGLSVPARRSAK)
Goatα NeuroD1	Santa Cruz, cat. no. sc-1086	Peptide sequence near C-terminus of mouse NeuroD1 (GSIFSSGAAAPRCEIPIDNI)
Mouseα CK14	Novocastra, cat. no. 2012–06	Peptide corresponding to the C-terminal 15 amino acids of human cytokeratin 14 conjugated to thyroglobulin
Goatα CK14	Santa Cruz, cat. no. sc-17104	Epitope mapping near C-terminus of human CK14
Rabbitα Sox9	Chemicon, AB5535	Affinity purified immunoglobulin raised against a synthetic peptide from Human Sox9.
Mouseα βIV-Tubulin	Sigma, cat. no.T7941, clone ONS. 1A6	Ascites fluid: synthetic peptide corresponding to the C-terminal sequence of β-tubulin isotype IV, conjugated to BSA
Mouseα CD54	R&D Systems, AF583	Recombinant extracellular domain from rat, affinity-purified (accession No. Q00238: amino acid residues Q28-T493)
Chickenα GFP	AbCam, ab13970	Recombinant full-length eGFP
Rabbitα GFP	AbCam, ab6556	Recombinant full-length eGFP
biotinylated Hamster anti-CD54 (b-CD54)	BD-Pharmingen, cat. no. 553251, clone 3E2	

**Table 2:** Methods for Antibody Staining used in this study.

IHC	Primary antibody (Table 1)	Secondary antibody	Tertiary step
Sox2+CK14	Sox2 (1:200)+CK14 (1:50)	b-DαR+Alexa488-DαGt	TSA →FITC-SA
Pax6+CK14	Pax6 (1:100)+CK14 (1:50)	b-DαM+Alexa488-DαGt	TSA →FITC-SA
Ascl1+CK14	Ascl1 (1:250)+CK14 (1:50)	b-DαM+Alexa488-DαGt	TSA →FITC-SA
Tuj1+CK14	Tuj1 (1:150)+CK14 (1:50)	Cy3-DαM+Alexa488-DαGt	
Sox9+Sox2+CK14	Sox9 (1:500)+Sox2 (1:40)+CK14 (1:50)	Alexa594-DαR+b-DαG+AMCA-DαM	Alexa488-SA
EdU+Sox9+CK14	EdU (as described in the instructions for the kit)	Sox9 (1:500)+CK14 (1:50)	Alexa594-DαR+AMCA-DαM
EdU+GFP	EdU (as described in the instructions for the kit)	GFP (1:1300)	Alexa488-GtαCh
PGP9.5+GFP	PGP9.5 (1:900)+GFP (1:1300)	Alexa594-DαR+Alexa488-GtαCh	
PGP9.5+CD54+GFP	PGP9.5 (1:900)+b-CD54 (1:100)+GFP (1:1300)	Alexa594-DαR+AMCA-SA+Alexa488-GtαCh	
Ascl1+GFP	Mash1 (1:2000)+GFP (1:1300)	b-DαM+Alexa488-GtαCh	TSA →Alexa594-SA
Neurog1+GFP	Neurog1 (1:1600)+GFP (1:1000)	b-DαGt+Alexa488-DαR	TSA →Alexa594-SA
NeuroD1+GFP	NeuroD1 (1:40)+GFP (1:1000)	b-DαGt+Alexa488-DαR	Alexa594-SA
β-gal+PGP9.5+GFP	β-gal (1:300)+PGP9.5 (1:900)+GFP(1:1300)	TxRd-DαGt+AMCA-DαR+Alexa488-GtαCh	
βIV-tubulin+PGP9.5+GFP	βIV-tubulin (1:300)+PGP9.5 (1:900)+GFP (1:1300)	TxRd-DαM+AMCA-DαR+Alexa488-GtαCh	
Pax6+PGP9.5+GFP	Pax6 (1:40)+PGP9.5 (1:900)+GFP (1:1300)	b-DαM	Alexa594-SA+AMCA-DαR +Alexa488-GtαCh
Pax6+CD54+GFP	Pax6 (1:40)+CD54 (1:100)+GFP (1:1000)	b-DαM	Alexa594-SA+AMCA-DαGt +Alexa488-DαR
Sox2	Sox2 (1:50)	b-DαGt	ABC →DAB

Detection of EdU was done following manufacturer's protocol. Briefly, Click-iT reagent containing buffer, CuSO4, Alexa Fluor azide, and buffer additive were mixed immediately before use, 100 μl of reagent was puddled on tissue sections and incubated for 30 minutes at room temperature, while protected from light. The sections were washed twice in 3% BSA in PBS prior to imaging.

### Quantitative Analysis

#### 3-week post-injury

Mouse OE infected with RRVs was sectioned completely and each section was analyzed for the presence of GFP-expressing clones. Subsequently, each clone was labeled by number, viral condition and location within the OE, i.e., turbinate number. Among clones each cell was counted and assigned a cell type, based on its morphology and location within the OE. GFP-labeled cells were identified as sustentacular cells (Sus) when their cell bodies were found at the apex of the epithelium and possessed a flat apical surface. OSNs were identified by their position directly below the Sus cells, as well as having an axon and a dendrite. Basal cells were identified as rounded, lacking a process and based on their physical location at the base of the epithelium. Duct/gland cells were identified as flattened, oriented as a chain of cells along the apical-basal axis, and/or extended deep to the basal lamina into the lamina propria. All clones were analyzed equivalently, across all viral conditions. For each viral condition, a minimum of three independent, infected OE tissues was analyzed.

#### 8-days post-injury

Mouse OE infected with RRVs was sectioned completely and sections were sampled from each condition, to analyze the expression of bHLH transcription factors. A survey of sections, at least 50 μm apart, was collected for each viral condition, in order to exclude double sampling from a single clone. Sections were initially selected on the basis that they possess at least one clone that contained round cells, situated basally and lack a neural process, i.e., GBC characteristics. Clones were then stained with antibodies against GFP along with Ascl1, Neurog1 and NeuroD1. Due to technical considerations it was not possible to analyze sections through the individual clones for more than a single factor at a time. In most circumstances all three sections/slide satisfied the requirements for analysis, in which case antibodies against each individual bHLH factor were applied to a separate section on the same slide. If all three sections did not contain qualifying samples, separate slides/sections were used to include additional samples for staining. bHLH-expressing cells were tallied by calculating the percentage of bHLH-expressing/GFP double-positive cells from among all GFP-expressing basal cells and neurons in a given sampled clone. A minimum of seven clones/OE was analyzed for each factor and each viral condition was analyzed in triplicate for bHLH factors. In total, a minimum of 27 clones/viral conditions was analyzed for each factor. Data were pooled for each bHLH factor/viral condition and statistical analysis was performed using Kruskal-Wallis, one-way non-parametric ANOVA on ranks and pairwise comparisons with Dunn’s methods; *p* values less than 0.05 were considered statistically significant.

### Photography/Image Processing

Sections were imaged with a Spot RT2 color digital camera attached to Nikon 800E microscope. Image preparation, assembly and analysis were performed in Adobe Photoshop. In the vast majority of images, only balance, contrast and evenness of the illumination were altered. On rare occasions, dust and debris that did not overlie the section were removed to satisfy aesthetic concerns.

### Statistics

All data was analyzed using either SigmaStat or R and a *p* value of 0.05 was used as the margin, below which data was considered statistically significant. To summarize overall procedures used in Figs 3–8 and 10, all data were first tested for normality using the Shapiro-Wilk Normality test where applicable. If the data passed normality, a standard parametric ANOVA test was performed to determine the validity of performing pairwise post-hoc Holm-Sidak tests. If the data failed the normality test, a non-parametric Kruskal-Wallis One-Way ANOVA on Ranks was used instead, followed preferably by post-hoc Conover-Iman, if allowed, because of its greater power, or by Dunn’s Method where the data failed the requirements of Conover-Iman. In two cases, Fig 5, effect of TFs on proliferation, and Fig 7, comparison of GBC progression, raw count data were necessarily converted to percentages. To perform multiple-testing and downstream post-hoc analysis on the data in Figs 5 and 7, data were first arcsin-transformed and then analyzed as described above. The data in Fig 6 neuronal suppression, in this case assayed by comparing clones that have or lack neurons, were analyzed using Fischer exact tests with Holm-Sidak multiple testing correction (similar to but more powerful than the Bonferroni multiple comparison tests for significance. Finally, for the data in Fig 8, the comparison of clone size between Sox2 cKO vs control allowed the use of the Mann-Whitney Rank Sum test. Each experiment was run in triplicate, at a minimum, and more replicates were performed to enhance statistical power in certain circumstances.

## Results

### Pax6 and Sox2 are near-universally expressed early during OE regeneration, but disappear upon neuronal differentiation

We examined the expression patterns of Pax6 and Sox2 during the regeneration of the mouse OE following inhalation of MeBr, which acts as a selectively olfactotoxic agent that kills OSNs and sustentacular cells and depletes other non-neuronal cell types. As has been described in rats, Pax6 and Sox2 are expressed in all spared cells of the lesioned OE at 1, 2 and 3 days after injury, with rare exception (Fig 2) [4]. As in rat, there is a progressive emergence of Pax6 (-) /Sox2 (-) GBCs and OSNs, marked by expression of neuron-specific tubulin (Tuj1), such that by 5 days post lesion the thick layer of Pax6 and Sox2-expressing cells is split into basal and apical (sustentacular somata) strata (Fig 2). As expected from the previous results, both the neuronal and non-neuronal populations are fully reconstituted over the following 6–8 weeks, likely as progeny from these spared Sox2/Pax6 (+) cells (data not shown) (2). Thus, the spared basal cells of lesioned OE, in aggregate, act as multipotent progenitors, ultimately giving rise to all cell types of the OE: sustentacular (Sus) cells, neurons, microvillar cells, duct/gland cells, GBCs, and HBCs [2,4,5,32,34].

The exclusion of Pax6 and Sox2 from neurons and the GBCs immediately upstream of them, suggests that down-regulation of the two factors may be required in order to allow neuron production to proceed. Thus, in order to investigate the functional role of Pax6 and Sox2 in more upstream GBCs, sustained expression of either *Pax6* or *Sox2* individually, or together, was accomplished via retroviral transduction with MuMoLV-derived retroviral vectors. In the case of the vector encoding both *Sox2* and *Pax6* (*SEP*), well-balanced expression of the two products was accomplished by inclusion of a viral 2A peptide sequence between the transcription factors (see Materials and Methods for the description of vector construction). Protein expression levels from the SEP construct were verified by Western blot (Fig 1) and compared to the reduced levels of the gene product that follow when an IERS motif separates *Sox2* and *Pax6* in a multicistronic construct in either orientation (data not shown). As a complement to our overexpression studies, *Sox2* was excised by genetic- or retroviral means to assess how elimination of Sox2 might alter neurogenesis.

### Globose basal cells, duct/gland cells and some horizontal basal cells are targets of retroviral infection following MeBr lesion

Within 24 hours following exposure to MeBr, the residual, spared cells at the base of the OE, which are responsible for regenerating the whole of the epithelium, become directly exposed to the nasal cavity and undergo enhanced proliferation. Consequently, the spared basal cells are susceptible to retroviral transduction for the first few days after MeBr exposure [5]. One day after MeBr lesion, when the retroviral vector is infused, the OE consists of residual HBCs (marked by strong expression of cytokeratins 5 and 14), duct/gland cells at or just superficial to the basal lamina (marked by the expression of Sox9), and spared GBCs (identified as neither HBCs nor duct cells, but expressing Sox2 and Pax6, in addition to having the simple morphology of GBCs) (Figs 2 and 3). Rarely, some triple labeled cells can be found (CK14 (+)/Sox2 (+)/Sox9 (+), which may represent a transition stage in the generation of duct/gland cells by HBCs in the MeBr—lesioned mouse OE [6] (Fig 3). Similarly, we occasionally observe other novel combinations at low frequency. For example, CK14 (+)/Sox2 (+)/Sox9 (+) basal cells (Fig 3) may represent HBCs that are transitioning into duct/gland cells [32,35]. Others that are CK14 (-) while expressing both Sox2 and Sox9 may represent GBCs giving rise to duct/gland cells [2].

Of these remaining cells types, the dividing cells, which are susceptible to retroviral integration, can be labeled via single intraperitoneal injection of EdU (data not shown) or by immunostaining with Ki-67 (Fig 3). At 1 day after MeBr lesion, most of the Ki-67-labeled cells are GBCs (CK14 (-)/Sox9 (-)) and duct/gland cells (CK14 (-)/Sox9 (+)); some HBCs (CK14 (+)/Sox9 (-)) are also dividing (Fig 3). Taken together, these data indicate that the potential targets for RVV infection in these studies include GBCs, duct/gland cells, and some HBCs.

### Sox2 makes the largest clones and Pax6 makes the smallest clones

We used retroviral vectors encoding *Sox2* and *Pax6* individually and together (*Sox2-E2A-Pax6*, abbreviated *SEP*) along with *eGFP*, and compared their effects to each other and to vector encoding *eGFP* alone (empty vector, abbreviated EV), in order to analyze the roles that Pax6 and Sox2 play during neurogenesis and epithelial regeneration (see Materials and Methods and Fig 1 for RVV maps). Epifluorescent examination of whole mounts harvested 3 weeks after nasal infusion reveals clusters of GFP (+) cells scattered across the turbinates and septal surfaces of the OE (Fig 4). On the order of 75 clusters were seen per infected mouse. By analogy to other experiments that use intranasal infusion to transduce cells [5], each cluster of cells most likely represents a clone of cells that takes its descent from a single infected progenitor for the following reasons: viral titer is low, the frequency of infection is relatively rare, the clones are widely distributed, and the expression of the marker protein is uniform within a cluster but can vary among clusters (indicating a locus of integration effect). Furthermore, when a mixture of two vectors encoding different markers is infused, each cluster is uniformly marked by a single label, indicating that each cluster is established by a single infective event [5]. The appearance of the clones in the whole mount can also be used to identify the predominant cell type(s) within it. When clones consist of Sus cells, the labeled cells can be identified as such by their superficial location and tight clustering, while neurons in the clones tend to be more dispersed and deep to the surface of the epithelium. On that basis, neurons are the predominant population within EV clones, which was verified upon analysis of cellular composition (see below).

Examination of the epithelial clones in whole mounts and counts of cell number per clone demonstrate that the Sox2-transduced clones, on average, are the largest; Pax6 are the smallest; and EV-transduced clones are larger than SEP clones (Sox2 > EV > SEP > Pax6, with averages of 72, 42, 23 and 13 cells/clone, respectively, Fig 4). When clones are sectioned and cells counted, the average number of cells per Sox2-transduced clone are greater than five times those with Pax6-transduction, three times those with SEP and almost twice those with EV (Kruskal-Wallis *p<* 0.05, Conover-Iman tests of multiple comparisons using rank sums, * *p* < 0.05) (Fig 4). Across all conditions and clones, Pax6-, SEP-, and EV-transduced clones top out at a maximum of 200 cells, though EV has one outlier that is 400 cells in number. In contrast, *Sox2*-transduced clones can be as large as 800 cells in number, with 20% of the clones counting greater than 200 cells/clone (Fig 4). A test for variance across conditions demonstrates that the distribution of Sox2 clone sizes is significantly different from all others when pairs were compared. Thus the expansion in the number of cells per clone due to the overexpression of *Sox2* is significantly attenuated when *Pax6* is co-expressed along with *Sox2*.

### Sox2 enhances cell proliferation early after infection

That overexpression of *Sox2* expands clone size relative to *Pax6*, both on its own and in SEP clones, suggests that Sox2 drives the proliferation of the progenitors in the regenerating OE in some manner. In some adult epithelial tissues, Sox2 has been associated with proliferation, but no such association has been demonstrated in neuronal tissue [36–38]. To assay whether transduction of *Sox2* enhances proliferation in the OE, animals were separately infected with one of four viral vectors and euthanized 8 days after infection, a time point during epithelial recovery from MeBr lesion by which robust neurogenesis has peaked. The mice were pulsed with EdU 2.5 hours prior to tissue harvest.

Across the four viral conditions, substantially greater mean numbers of EdU (+)/GFP (+) cells are found in *Sox2*-transduced clones, compared to EV (One-Way ANOVA on Ranks performed on arcsin-transformed percentages, p < 0.05)(Fig 5). However, by comparison with EV there was no statistically significant difference with any of the other three conditions. These data suggest that Sox2 directly or indirectly influences cell cycle genes to increase proliferation among transduced olfactory progenitors.

### Compositional analysis of transduced clones

Sections through the transduced clones were stained for GFP and the neuronal marker PGP9.5. As a consequence, clone composition was determined on the basis of immunochemical profile, epithelial position, and morphology as follows: basal cells are found below the band of PGP9.5 (+) neurons; Sus cells are above; and duct/gland cells are identified by forming a line of cells passing through the epithelium to reach the epithelial surface. The typical clone derived from infection with EV is composed of neurons, some Sus cells, and occasionally a few basal cells (Fig 6). Other clones from EV conditions are composed exclusively of Sus cells or neurons.

By comparison with EV, transduction with Pax6 less often produced clones that included neurons (Fig 6J); for the EV and Pax6 constructs as well as the Sox2 and SEP vectors described below, clones that lack neurons are composed mainly of Sus cells with occasional duct/gland and basal cells (Fig 6E and 6F), and seldom of duct/gland cells only (Fig 6G). To get a clearer picture of Pax6’s effects on neuronal suppression, we analyzed a large pool of clones (EV, *n* = 56; Pax6, *n* = 151) from a total of three separate animals for each vector and counted the number of clones that have OSNs vs. those that don’t (Fig 6). Whereas greater than 85% of the EV clones contain neurons, less than 50% of Pax6-infected clones have them (Fisher Exact, *p <* 0.002)(Fig 6J). Pax6-transduced clones lacking neurons can consist of basal cells, which lay below the band of stained neurons, and duct/gland cells that extend from the lamina propria through the basal lamina and traverse the height of the epithelium (Fig 6C and 6G). The suppression of neurogenesis is also evident both by comparing the median number of neurons per Pax6 clone vs. EV (0 vs. 8, respectively; Fig 6I), as well as the average (8.0 vs. 31.5) or range of neuron numbers as indicated by the 90^th^ percentile of the distribution (Fig 6I).

Previous studies have shown that Sox2 can serve as a co-factor along with Pax6 in regulating gene transcription (for example, in the case of the δ-crystallin gene in lens fiber cells; [39]. Given their co-expression in GBCs and HBCs of the OE, we tested whether the addition of a potential co-factor, namely Sox2, would cooperatively function to completely suppress the formation of neurons within the clone. In contrast to Pax6 alone, typical SEP clones more often contain neurons, Sus cells, and occasionally basal cells (Fig 6I and 6J). Thus, upon the co-transduction with Sox2 and Pax6 the percentage of clones that include neurons increased to 71% (*n* = 165), a substantial increase compared to Pax6 and close to the effects of Sox2 by itself (Fisher Exact *p* < 0.05, Fig 6J). The SEP-transduced clones vary substantially in size and complexity (for example, possessing both neurons and Sus cells); clonal composition of SEP-transduced clones appears to be less like Pax6-transduced clones and more like the EV condition (Fig 6), though clone size is smaller (Fig 4). Even though neurons are more common constituents of SEP-transduced clones as compared to Pax6 alone, nevertheless, SEP transduction continues to suppress the production of neurons relative to EV as indicated by the median number of neurons per clone (3 vs. 8, respectively), as well as the average (18.4 vs. 31.5, respectively) or range of neuron number as indicated by the 90^th^ percentile of the distribution (Fig 6E). However, it is worth noting the outliers in the distribution of neuron number in SEP clones, which are markedly greater than in the EV clones. These data suggest that Sox2 partially relieves the suppressive effects of Pax6 on the production of neurons.

That Sox2 counteracts rather than reinforces the suppression of neurogenesis caused by Pax6 is opposite to a role that has been ascribed to Sox2 in the central nervous system, namely inhibiting the formation and differentiation of neurons [22,40,41]. Additionally, combining Sox2 and Pax6 did not have synergistic effects, in contrast to the cooperative behavior observed when Pax6-Sox2 bind and transactivate δ-crystallin gene expression in the developing lens [42]. Accordingly, we tested the effect of transduction with Sox2 alone with respect to clone size and composition. In at least partial concordance with previous observations, the percentage of neuron-containing clones (66% out of a total of 118 analyzed) remains suppressed relative to EV (86% as noted above; Fischer Exact, *p <* 0.05), and the median number of neurons per Sox2 clone remains less than in EV (2.5 vs. 8) (Fig 6I and 6J). Indeed, the non-neuron-containing Sox2-transduced clones are composed of only Sus cells for the most part (Fig 6F), a result that is certainly in line with previous reports of the effect of Sox2 overexpression in the developing CNS. However, the percentage of clones with neurons is distinctly greater than Pax6 alone and roughly on par with SEP transduction (Fig 6J). Moreover, when the number of neurons per clone is analyzed, a substantial group of Sox2-transduced clones contain a very large number of neurons, i.e., 12% of Sox2-transduced neuron-generating clones have in excess of 200 OSNs per clone—a much higher percentage than with EV, Pax6, or SEP transduction; the difference is also evident when comparing the average numbers of neurons per clone (for Sox2, EV, Pax6 and SEP– 61.5 vs. 31.5 vs. 8.0 vs. 18.4, respectively) and the 90^th^ percentiles of the several distributions (Fig 6I). These data suggest that Sox2 and Pax6 both suppress the production of neurons incompletely, while Sox2 on its own, if the block to neurogenesis is overcome, leads to a more sustained increase in proliferation and greatly enhanced neuron production but without any overall increase in the formation of Sus cells, basal cells, or D/G cells (Fig 6).

With respect to the other epithelial cell types, both of the transduced transcription factors had additional demonstrable effects. Pax6, as well as suppressing the formation of neurons, caused a modest, but statistically significant increase—to nearly 10%–in the average number of duct/gland cells relative to the EV control (Kruskal-Wallis, *p <* 0.001) (Fig 6G and 6I). Conversely, the average number of Sus cells is decreased with Pax6 transduction, relative to EV (Fig 6I). These outcomes are not unexpected, given the expression of Pax6 without Sox2 in the gland/duct cells of the olfactory epithelium [4]. Finally, Sox2 transduction had no discernable effect on the number of basal cells relative to EV, while the generation of basal cells is suppressed by co-transduction with Pax6 in the SEP vector (Fig 6I).

### Viral transduction shifts the composition of the GBC sub-populations in the regenerating epithelium

Because neurogenesis is the process most profoundly affected by the Sox2 vector, we assessed the expression patterns of key neural progenitor cell-associated transcription factors after transduction. As noted above, a well-established cascade of basic helix-loop-helix (bHLH) transcription factors can be used to identify specific stages in the hierarchy of these neuronal progenitors: Ascl1 (Mash1) is purported to label transit amplifying cells, while Neurog1 and NeuroD1 commonly co-label the population of immediate neuronal precursors although Neurog1 expression may slightly lead that of NeuroD1 (Fig 7) [7–9,33,43,44]. Using specific antibodies for each of the bHLH factors, we characterized the GFP-expressing clones at 8 days post infection, which is a time when the recovering epithelium is still dominated by the expanded progenitor population with a more limited restoration of the neuronal population. Ascl1-, Neurog1-, and NeuroD1-immunoreactive progenitors are all present globally within the OE at this time point, and in some but not all of the clones transduced with each of the four vectors. Notably, the percent of transcription factor-positive clones varies significantly as a function of vector across the four vectors (Chi-square for Ascl1-containing clones = 20.42, p < 0.001; for Neurog1-containing clones = 8.28, p < 0.05; for NeuroD1-containing clones = 10.50, p < 0.05) (Fig 7Q).

The influence of an individual vector on clonal composition was analyzed with respect to each of the bHLH transcription factors by determining the percent of immunopositive cells for each individual bHLH factor (Fig 7N–7P). The nature of the viral vector had a statistically significant effect on the percentage of each GBC type per clone (Kruskal-Wallis for Ascl1 (+), Neurog1 (+) and NeuroD1 (+) GBCs: H = 55.79, p < 0.001; H = 26.42, p < 0.001; H = 36.14, p < 0.001, respectively).

By comparison with EV, Sox2 transduction produced clones that more often contained Ascl1 (+), Neurog1 (+), and NeuroD1 (+) cells (Fig 7Q) and in which each of the three types of GBCs were more abundant (Fig 7N–7P) (Dunn’s method for pairwise comparison, Q = 5.810, 4.468, 5.209, respectively; all reach statistical significance at p < 0.05). In contrast, Pax6 transduction did not lead to an expansion in the population of Ascl1 (+) GBCs as measured either by the abundance within clones (Fig 7N–7P) or by the number of clones that contained Ascl1 (+) GBCs (Fig 7Q). The Pax6 vector but did lead to an increase in Neurog1 (+) and NeuroD1 (+) cells (Q = 3.218, 4.782, respectively; significant at the level of p < 0.05). The “rightward” shift pushes the population toward GBCs that are further downstream in the hierarchy. When transduced with both Sox2 and Pax6, the number of Ascl1 (+) cells remains high and statistically significant (Q = 3.758, p < 0.05) by comparison with EV, but the abundance of Neurog1 (+) and NeuroD1 (+) GBCs, while greater than EV, are no longer significantly different from the observations following EV transduction. These data suggest that introducing Sox2 enhances the expression of Ascl1, which serves as a molecular signature for highly proliferating, transit-amplifying progenitors that are capable of producing multitudes of neurons; an effect that Sox2 elicits after eight or more days of sustained Ascl1 expression. On the other hand, Pax6 has the effect of depleting the overall numbers or reducing the percentage of clones that contain Ascl1, suggesting that neuronal progenitors are being pushed to advance past the transit-amplifying stage, thereby limiting their numbers and causing neuronal clones to be smaller in size after three weeks of sustained expression. When both Sox2 and Pax6 are overexpressed the effect more closely resembles that of Sox2 alone, but is intermediate, suggesting that the presence of both factors maintains a more immature state rather than pushing through to the production of neurons.

### Conditional deletion of Sox2 by RV transduction reduces clone size and attenuates neuronal production

The altered composition of the clones following transduction with the Sox2-encoding retrovirus suggests that Sox2 may play two roles in the neurogenic process, at some, probably early, point suppressing the commitment to the neuronal lineage and at other, probably later, points enhancing neuronal birth and/or differentiation. The availability of a *floxed-Sox2* mouse offers the opportunity for conditional deletion of the *Sox2* open reading frame either by transducing with a Cre recombinase-encoded RVV, *Cre-IRES-eGFP* (Fig 8A), or by means of a cell-type specific expression of inducible Cre recombinase (see the following section). These manipulations allow us to examine the role of Sox2 at the clonal level, leaving most of the progenitor cells intact and unaffected. The fate of the *Sox2*-deleted progeny was traced by activation of *LacZ* expression in the transduced, *Sox2*-deleted cells by genetic recombination of the *R26*^*fl(stop)LacZ*^ locus in bigenic *Sox2*^*flox/flox*^;*R26*^*fl(stop)LacZ*^ (SR) indicator mice.

Deletion of *Sox2* following transduction of SR mice with Cre recombinase typically results in smaller, well-isolated clones of β-galactosidase- and GFP-expressing cells, which may be composed of one or only a few cells (Fig 8C), in contrast to transduction of *R26*^*fl(stop)LacZ*^ indicator mice, which gives rise to markedly larger clones (Fig 8B). Cell numbers in the clones found in floxed *Sox2* mice are significantly reduced averaging about 20% of the numbers observed in the control (Mann-Whitney-U test, *p* < 0.001) (Fig 8H). In sections stained for GFP (to mark cells expressing Cre), β-gal (to identify recombination), and PGP9.5 (a neuronal marker), i.e., clones in which there has been recombination and deletion of Sox2, the clones contain few, if any marker-positive neurons (Fig 8E–8G). In contrast, clones identified by recombination in the control indicator mice contain more neurons on average than in the floxed *Sox2* mice (Fig 8D). Many of the clones in the *Sox2* conditional knockout (cKO) mice are composed exclusively of Sus cells in singletons or pairs, in their usual location at the apical margin of the epithelium (e.g., Fig 8F). These apical cells are demonstrably Sus cells: they sit superficial to PGP9.5 (+) neurons, and they do not stain with the respiratory epithelial maker, β-IV-tubulin (Fig 8F). In addition, we see GFP-labeled HBCs following Sox2 deletion, as shown by labeling either by position and morphology (Fig 8G) or staining with CD54 (an HBC-specific marker). However, GBCs, which are neither CD54 (+) nor PGP9.5 (+), are markedly less frequent (data not shown).

The effect on neuronal production was analyzed across the larger pool of clones found in serial sections through the olfactory area of three *Sox2* cKO mice vs. three control mice (*n* = 243 and 135 clones, respectively). A greater number of *Cre*-transduced clones in the *Sox2*-cKO mice lack neurons, compared to control mice (60% vs. 25%, respectively)(Fischer Exact test, *p* < 0.001) (Fig 8I). The level of neuronal suppression with recombination is similar to that observed following transduction with Pax6 by itself (cf. Fig 6), suggesting that Pax6 acts in isolation, as a consequence of the elimination of Sox2. In this scenario, Pax6, when acting alone at presumed endogenous levels of expression, suppresses neurogenesis to roughly the same degree as caused by transduction and maintained expression. Among the few clones that generated and retained neurons, *Sox2* cKO clones contain, on average, 1/10^th^ the number of neurons of control clones (Kruskal-Wallis, *p <* 0.001, Fig 8J); Sus cells are also significantly fewer in the *Sox2* cKO clones, as compared to control (Fig 8J). In contrast, the average number of basal cells are more numerous in the *Sox2* cKO condition, compared to control, although the difference does not reach statistical significance (Fig 8I). These data suggest that the differentiation of neurons and Sus cells following KO of Sox2 is reduced, seemingly accompanied by a slight expansion in the number of basal cells. It is worth noting that Pax6 labeling remains evident in HBCs and Sus cells of *Sox2*-cKO olfactory tissue, and the staining intensity is equivalent to that in these cell types in the surrounding, non-recombined, regenerating epithelium (Fig 9). These data suggest that Pax6 expression levels do not depend on Sox2 expression, in contrast to the dependency observed in the retina [45]. In sum, *Sox2* is not apparently necessary for neuronal production *per se*; however, Sox2 apparently plays a significant role in the expansion of neuronal progenitor cells.

### Conditional deletion of Sox2 in HBCs also reduces clone size and attenuates neuronal production

For the reasons described above (in the section introducing the transduction approach), the basal cells spared by MeBr lesion and thereby subject to retroviral vector infection 1-day post-exposure and subsequent transduction with Cre recombinase are likely to be a heterogeneous mix of HBCs, GBCs and duct/gland cells. However, HBC-specific, Cre-mediated recombination can be accomplished robustly in advance of MeBr lesion when a *Keratin5* (*K5*) promoter is used to drive a tamoxifen-dependent version of the recombinase (*K5*^*CreERT2*^). Such an approach provides a greater degree of surety as to when the gene has been eliminated than can be achieved by retroviral delivery of Cre [6]. Subsequent to epithelial damage, HBCs activate from dormancy as a consequence of the down-regulation of p63, and HBC-derived GBCs then appear and give rise to the same panoply of cell types as derive from stem-like GBCs [6,46]. In particular, neurogenesis does not occur following injury unless GBCs are spared or regenerated [47]. As a consequence, the effects of *Sox2* knockout by recombination can be assigned to the most “upstream” stage of the GBC neurogenic hierarchy (Fig 7).

Parental *Sox2*^*flox/flox*^; *R26*^*fl(stop)fl(stop)-LacZ*^ mice were bred to *K5*^*CreERT2*^ mice in order to generate a trigenic KSR line bringing together driver and indicator alleles and either one or both copies of the floxed Sox2 allele. Mice of the desired genotypes were injected IP with 300 mg/kg tamoxifen and either exposed to MeBr two weeks later or left unlesioned. In unlesioned mice that were of either genotype, the β-galactosidase (+) recombined cells remain within the monolayer of HBCs that sit apposed to the basal lamina (Fig 10A and 10C, respectively); in particular, the HBCs appear unaffected by the demonstrable absence of Sox2 (Fig 10C and 10C’). In animals exposed to MeBr, the recombined cells showed evidence of activation independent of genotype (Fig 10B and 10D). However, in those clusters of labeled cells that we presume to be clonal because they sit well isolated within a broad swathe of the OE that lacks recombination, the average size of the clones is smaller overall in the homozygous *Sox2*^*flox/flox*^ mice as compared to heterozygous and wild type mice (Fig 9E and data not shown). In addition, the outcome of activation with respect to cell types that are generated is also markedly different (Figs 10B vs. 9D for mice that are floxed-Sox2 heterozygotes vs. homozygotes, respectively). In Tam-treated, lesioned-recovered homozygous *Sox2*^*flox/flox*^ mice, a lower percentage of β-gal (+) neurons and a higher percentage of HBCs are observed when summed across all recombined cells independent of clonality as compared to heterozygous mice (Fig 10F; Holm-Sidak method for multiple comparisons, t = 5.68, p < 0.001 and t = 4.72, p < 0.001, for neurons and HBCs, respectively). These data suggest that the elimination of Sox2 does not prevent the generation of GBCs from HBCs after tissue damage, but does cause a significant reduction in the number and percentage of neurons that are produced, confirming the effects observed following retroviral transduction with Cre, as well as a relative enhancement in the percentage of HBCs, although the latter can be explained almost completely by the decrease in clonal size overall.

## Discussion

The current work demonstrates that overexpression of Sox2 or Pax6 in progenitor cells of the regenerating OE suppresses neurogenesis by comparison with EV, as fewer clones contain neurons. Among clones that escape suppression and generate neurons, Pax6 transduction effectively reduces neuron numbers, compared to EV, whereas Sox2 transduction increases their numbers. Near-equimolar co-transduction with both factors (SEP) leads to an intermediate effect; the suppressive effects on neurogenesis are similar to Sox2 transduction, while among neural clones, neuron numbers fall between the values for Sox2 and Pax6. The expansion of neuron numbers at three weeks following Sox2 transduction is foreshadowed by increased GBC proliferation and enrichment in GBCs expressing bHLH transcription factors at 8 days, particularly in those marked by Ascl1. Conversely, elimination of Sox2 by genetic excision leads to small clones with few neurons, whether via retroviral transduction or the HBC-specific *K5* promoter-driven Cre.

The constellation of effects from all three retroviral vectors raises the issues of which cell type(s) is/are actually being infected and when expression of the transduced genes becomes functional. We show that HBCs, duct/gland cells, and GBCs are all proliferating and accessible for infection at one-day post-lesion in the mouse OE. Among those cell types, GBCs are the most commonly infected, judging by the cellular composition among clones (containing neurons and non-neural cells, together or separately) [2], as well as results from genetic lineage tracing [48]. The other commonly infected cell type—duct/gland cells—gives rise only to themselves and Sus cells [2,5]. Infection of HBCs rarely occurs in rats, but even in their multi-lineage capacity, HBCs apparently differentiate into GBCs before generating neurons and likely the other OE cell types [6,46,49]. Thus, the primary targets for transduction are GBCs, and duct/gland cells.

With respect to the expression kinetics of these exogenous factors, we know that GFP is detectable in OE whole mounts 2 days after infection (data not shown). Additionally, retroviral transduction achieves maximal and sustained expression by 48 hours (49), such that detectable labeling for Sox2 and Pax6 is seen in transduced OSNs 3 weeks later (data not shown). On that basis, we suggest that expression of the exogenous factors initially coincides with endogenous expression of Sox2 and Pax6, either slightly in advance of, or in concert with Ascl1 (based on their expression patterns following MeBr lesion). Subsequently, endogenous Pax6 and Sox2 levels decline as neurogenesis proceeds, and are rarely seen in Neurog1- and NeuroD1-expressing GBCs [4].

Thus, the effects of viral transduction need to be considered within a complex matrix: stage within the progenitor cell hierarchy when exogenous levels eclipse the endogenous concentrations and the interaction between the transcription factors as driven by their relative levels. While the dynamics of regeneration and of RVV transduction add some complexity to the analysis, the current experimental approach allows one to appreciate the full range of roles that these transcription factors are playing within the OE. The heterogeneity of outcomes is consistent with the interaction between transcription factors and the multitude of signals that are impinging on the progenitor cells within the environment of the regenerating OE. As such, the transcription factors singly or together are largely insufficient in producing an all or nothing outcome.

The effects of Pax6, in our studies, are consistent with findings in the retina, where it antagonizes the production of neurons and biases fate toward the production of non-neuronal cells (duct/gland cells in our case)[50]. Likewise, in neuron-containing clones in the OE, far fewer neurons/clone are generated than in all other viral conditions, a result that may reflect the “rightward” shift in the GBC population to ones expressing bHLH transcription factors—namely Neurog1 and NeuroD1 –found more downstream in the neurogenic hierarchy. This outcome is in agreement with previous over-expression studies, where high levels of Pax6 accelerate neurogenesis, thereby reducing the number of neurons produced [11,51]. In the cerebral cortex, Pax6 was found to directly bind to the *Sox9* promoter [52], and in the OE Pax6 is co-expressed with Sox9 (but not Sox2) in duct/gland cells, providing a potential mechanism by which Pax6 enriches for duct/gland cells, here.

On the other hand, the bi-functional role of Sox2 during olfactory neurogenesis (suppressing the number of neuron-containing clones but increasing the numbers of neurons per clone when neurogenesis proceeds) fits with the complex roles played by Sox2 in the many tissues that express it. Originally described as a suppressor of neurogenesis [22,41], the relative overproduction of olfactory neurons in some clones may represent an effect imposed after neurogenesis has been initiated/has escaped from that suppressive effect. We favor the scenario that transit-amplifying neuronal-progenitors, which are marked by Ascl1 expression, are the locus for this pro-neurogenic effect. In agreement with this, Ascl1-expressing GBCs express Sox2 and Pax6, albeit at somewhat reduced levels, in the normal and lesioned-recovering epithelium and are significantly increased when Sox2 is introduced as shown here [4]. Additionally, Sox2 plays a role in fostering proliferation based on both the increased EdU labeling (and clone size) with overexpression and the diminished clone size upon genetic excision. Furthermore, evidence suggests that Ascl1-expressing GBCs undergo amplification by symmetric division [9,53]. Together, these data predict that sustained proliferation of Ascl1-expressing GBCs will increase the production of neurons, just like we observe among Sox2-transduced clones. In support of our findings, Sox2 increases Ascl1 expression and amplifies progenitor cell offspring elsewhere, including the fetal lung and OE [38,54].

We must also take into account that the effects of introducing our exogenous factors occur within a setting where Pax6 and/or Sox2 are already being expressed. Therefore, the relative levels of Pax6 and Sox2 need to be considered, since each factor has demonstrated functional roles that differ by their dosage [12,17,21,52]. Of note, Pax6 and Sox2 regulate the relative levels of each other’s expression in the retina, in both directions [50] and in the OE where Pax6 acts on Sox2 [16,50]. Furthermore, within the OE, Pax6 and Sox2 are expressed at lower levels among HBCs/GBCs and at highest levels in Sus cells; Pax6, by itself, is expressed in duct/gland cells [4]. As such, the variety of cellular outcomes that we observe here can easily be explained by modulation in the relative levels of Pax6 and/or Sox2 within different progenitor cell types.

Our results with Sox2 loss-of-function complement our Pax6 gain-of-function studies, both of which suppress neurogenesis. In both cases, the numbers of neuron-containing clones are reduced, as are the number of neurons that are generated when suppression is overcome. Together, these findings suggest that Sox2 and Pax6 counteract each other, to maintain balance in each other’s levels and subsequent influences on cell fate—with an emphasis on neuronal vs. non-neuronal outcomes. Similarly, regeneration is impaired when Pax6 is eliminated among HBCs, prior to injury, which results in a reduction in neurogenesis and of duct/gland cell formation [16]. Given that these defects are borne from HBCs and the GBCs derived from them, the reduction in duct/gland cells supports our hypothesis that Pax6 is an important regulator of duct/gland cell fate. Furthermore, the aberrant increase in Sox2 expression concomitant with suppression of neurogenesis from conditional loss of Pax6 [16] supports our hypothesis that Sox2 and Pax6 counteract each other’s roles in the OE and that neural suppression by Sox2 is upstream of its pro-neurogenic effects on transit-amplifying GBCs.

Another parameter that can influence the activity of Pax6 and Sox2 are their binding partners. Most notably, Pax6 and Sox2 are known to physically interact and cooperatively activate gene expression in the lens [42], while in the retina they antagonize each other’s role [50]. Likewise, the cooperative interaction of Pax6 and Sox2 in regulating gene expression, as shown by their binding to the same promoter and enhancer regions is common behavior at the loci of many genes involved in neurogenesis [55]. Several other transcription factors are known to partner with Sox2, including members of the POU family of TFs [56,57], which cooperatively regulate Pax6 in the olfactory placode [58,59]. Thus, the nature of the molecular interactions among Sox2, Pax6, and other, as yet unidentified transcription factors may dictate whether Sox2 and Pax6 cooperate or antagonize each other when regulating downstream genes. Certainly, the data presented here give evidence that the two transcription factors can exert both similar and opposite effects.

In summary, Pax6 and Sox2 demonstrate reciprocal interactions in regulating neurogenesis among progenitors in the olfactory epithelium, with Sox2 selectively expanding the neuronal population, likely by enhancing the proliferation and expansion of the transit amplifying, Ascl1-expressing GBC progenitor. The yin-yang effects of these two factors in the OE does not necessarily contradict the standard model, wherein they cooperate in regulating their target genes, since their counteractive interactions are aimed at achieving a competitive balance. As potent similarities exist between our finding here and the roles of Pax6 and Sox2 in other neurogenic matrices, future work in the olfactory epithelium will prove valuable in dissecting the nature of their respective and joint roles in neuronal stem and progenitor cells more broadly.
